# Supplementary material for: A fault-intrusion-tolerant system and deadline-aware algorithm for scheduling scientific workflow in the cloud
Source: PeerJ Comput Sci. 2021 Nov 2;7:e747. doi: 10.7717/peerj-cs.747 (PMC8576554; doi:10.7717/peerj-cs.747)
Supplement: Supplemental Information 6 [file peerj-cs-07-747-s006.zip › Myproject1.1/docs/org/workflowsim/clustering/balancing/methods/ChildAwareHorizontalClustering.html]

ChildAwareHorizontalClustering


JavaScript is disabled on your browser.


- Overview
- Package
- Class
- Use
- Tree
- Deprecated
- Index
- Help

- Prev Class
- Next Class

- Frames
- No Frames

- All Classes

- Summary:
- Nested |
- Field |
- Constr |
- Method

- Detail:
- Field |
- Constr |
- Method


org.workflowsim.clustering.balancing.methods

## Class ChildAwareHorizontalClustering

- java.lang.Object
- - org.workflowsim.clustering.balancing.methods.BalancingMethod
  - - org.workflowsim.clustering.balancing.methods.ChildAwareHorizontalClustering

- ---

    

  ```
  public class ChildAwareHorizontalClustering
  extends BalancingMethod
  ```

  ChildAwareHorizontalClsutering is a method that merges a task and its children

  Since:
  :   WorkflowSim Toolkit 1.0

- - ### Constructor Summary

    Constructors

    Constructor and Description

    `ChildAwareHorizontalClustering(java.util.Map levelMap,
    java.util.Map taskMap,
    int clusterNum)`

    Initialize a ChildAwareHorizontalClustering object
  - ### Method Summary

    Methods

    | Modifier and Type | Method and Description |
    | --- | --- |
    | `void` | `run()` The main function |

    - ### Methods inherited from class org.workflowsim.clustering.balancing.methods.BalancingMethod

      `addTaskSet2TaskSet, cleanTaskSetChecked, getClusterNum, getLevelMap, getTaskMap`
    - ### Methods inherited from class java.lang.Object

      `clone, equals, finalize, getClass, hashCode, notify, notifyAll, toString, wait, wait, wait`

- - ### Constructor Detail


    - #### ChildAwareHorizontalClustering

      ```
      public ChildAwareHorizontalClustering(java.util.Map levelMap,
                                    java.util.Map taskMap,
                                    int clusterNum)
      ```

      Initialize a ChildAwareHorizontalClustering object

      Parameters:
      :   `levelMap` - the level map
      :   `taskMap` - the task map
      :   `clusterNum` - the clusters.num
  - ### Method Detail


    - #### run

      ```
      public void run()
      ```

      The main function

      **Overrides:**
      :   `run` in class `BalancingMethod`


- Overview
- Package
- Class
- Use
- Tree
- Deprecated
- Index
- Help

- Prev Class
- Next Class

- Frames
- No Frames

- All Classes

- Summary:
- Nested |
- Field |
- Constr |
- Method

- Detail:
- Field |
- Constr |
- Method
